# Unmet Needs for a Rapid Diagnosis of Chikungunya Virus Infection

**DOI:** 10.3201/eid2210.151784

**Published:** 2016-10

**Authors:** Elisa Burdino, Guido Calleri, Pietro Caramello, Valeria Ghisetti

**Affiliations:** Amedeo di Savoia Hospital, Torino, Italy

**Keywords:** chikungunya, chikungunya virus infection, diagnosis, point-of-care assays, infections in travelers, rapid test, laboratory diagnosis, viruses, acute phase, test sensitivity, test specificity, epidemiology, outbreak control, differential diagnosis, travel-associated infection, imported cases, vector-borne infections

## Abstract

Rapid Diagnosis of Chikungunya Virus Infection

**To the Editor**: Chikungunya virus (CHIKV) has become a global health problem. Clinical manifestations are not specific and are difficult to differentiate from those of similar viral diseases (e.g., dengue and Zika virus disease). Diagnostic laboratories must be prepared to meet the changing epidemiology of viral diseases. CHIKV infection is currently identified by viral genome detection, using reverse transcription PCR (RT-PCR), viral culture, and serologic testing for IgG and IgM by indirect immunofluorescence (IFA) or ELISA. RT-PCR is most sensitive during the early phase of CHIKV infection (within 5–7 days of symptom onset), but its use is limited by the short viremic phase of the disease. After the acute phase, serologic testing for IgG and IgM is a more accurate indicator of disease. Molecular and serologic tests are complementary, reliable, and sensitive methods, but they require special equipment and a medium-to-high level of technical skill that may not be available in many laboratories, especially those in rural areas, where outbreaks usually occur.

Accurate and rapid detection of CHIKV infection by reliable point-of-care (POC) assays has been recommended to facilitate outbreak control. To meet this need, rapid CHIKV IgM POC tests are now available, but little information exists regarding their performance. The sensitivity of these tests evaluated in settings with a high prevalence of CHIKV infection is poor (range 1.9%–50.8%) compared with that for reference assays, especially in the acute phase of disease (*1*–*5*). In low-prevalence settings, CHIKV infection generally occurs as imported cases in travelers returning from disease-endemic countries. Diagnosis of such cases requires discrimination between CHIKV, dengue, Zika, and other febrile diseases in the differential diagnosis; this discrimination could be facilitated by the use of a reliable POC assay. The recent Zika virus disease outbreak in South America also highlights the worldwide need for rapid reliable POC tests.

From June 2014 through November 2015, eight patients who had returned to Italy from the Caribbean and Latin America were referred to the regional Center for Infectious Diseases, Amedeo di Savoia Hospital, in Turin for travel-associated CHIKV infection. These cases were the first in the region after 3 years without imported cases. We used IFA (Euroimmun AG, Lubek, Germany) and real-time RT-PCR (TIB MOLBIOL GmbH, Berlin, Germany) for CHIKV diagnosis. In addition, we evaluated the OnSite Chikungunya IgM Combo Rapid Test CE (CTK Biotech, San Diego, CA, USA) for CHIKV infection.

The rapid test identified IgM in only 3 of 8 patients (sensitivity 37.5%). All patients were negative for viral RNA, probably due to the time elapsed between symptom onset and serum sample collection, as confirmed by the presence of CHIKV IgG in most patients. No false-positive or invalid results were recorded with the rapid test on 30 CHIKV-negative serum samples (specificity 100%; positive and negative predictive values 37.5% and 100%, respectively).

Rapid and appropriate diagnostic tools are needed to slow or stop the worldwide spread of CHIKV. Rapid POC tests are highly cost-effective because they are easy to perform and can be disseminated to many laboratories for differentiating between diseases that are similar. Moreover, their results can easily be evaluated and shared within networks of reference laboratories.

However, our findings, in agreement with those of others, show that current rapid CHIKV tests perform poorly and need major improvement (Table) (*1*–*5*). This poor performance might have several explanations. For example, CHIKV patients do not often seek medical care in the early course of the disease. Most patients in our study were no longer in the acute phase of illness: the diagnosis was made a mean of 16.8 (range 7–30) days after fever onset, and when tested, all patients were viral RNA–negative by real-time RT-PCR. POC reactivity generally increases in patients with illness duration of >1 week (*1*–*5*), but this was not the case in our study. Genetic differences in circulating CHIKV lineages could also explain poor testing performance. Furthermore, the OnSite Chikungunya IgM Combo CE POC test uses a recombinant antigen covering the 226 residues of the E1 gene from CHIKV variant A226; recent studies on CHIKV protein characterization showed that more sensitive serologic assays can be obtained using specific early-phase E2 glycoprotein as antigens (*3*). 

**Table:** Reported sensitivity and specificity of rapid point-of-care tests for detecting chikungunya virus, 2008–2015*

Reference and test(s)	Time from symptom onset to testing, d	Sensitivity, %‡	Specificity, %‡	Test reference standard
(*1*)				
OnSite Chikungunya IgM Rapid Test	1 to >21	20.5	100	Capture ELISA IgM (in house) with Asian lineage virus; rRT-PCR
SD BIOLINE Chikungunya IgM test	1 to >21	50.8	89.2	Capture ELISA IgM (in house) with Asian lineage virus; rRT-PCR
(*2*)				
SD BIOLINE Chikungunya IgM test	<7; 8 to >14§	22; 83	88; 71	ELISA IgM; rRT-PCR
(*3*)				
OnSite Chikungunya IgM Rapid Test	3.75 to >7	12.1	100	IgM IFA; capture ELISA IgM (in house); rRT-PCR
(*4*)				
SD BIOLINE Chikungunya IgM test	3–8	1.9–3.9	92.5–95.0	Capture ELISA IgM; rRT-PCR
(*5*)				
OnSite Chikungunya IgM Combo Rapid Test CE	NA	20	93	Capture ELISA IgM/IgG (in house); plaque reduction neutralization test
SD BIOLINE Chikungunya IgM test	NA	30	73	Capture ELISA IgM/IgG (in house); plaque reduction neutralization test
This study				
OnSite Chikungunya IgM Combo Rapid Test CE	7 to 30	37.5	100	IFA IgM/IgG (commercial); rRT-PCR

*IFA, indirect immunofluorescence assay; NA, not applicable; rRT-PCR, real-time reverse transcription PCR. †Manufacturers: CTK Biotech, San Diego, CA, USA (OnSite Chikungunya IgM Combo Rapid Test CE and OnSite Chikungunya IgM Rapid Test); Standard Diagnostics, Inc., Seoul, South Korea (SD BIOLINE Chikungunya IgM test). ‡Values are those reported in the original publications. §Testing was done at 2 different time points after symptom onset.

The successful use of rapid immunochromatography-based assays with monoclonal antibodies to detect viral diseases (e.g., dengue) has encouraged the development of rapid immunoassays for CHIKV antigens, and preliminary results for these assays seem promising (*6*). External quality assessment programs for POC tests and quality controls consisting of standardized positive serum could also be helpful for improving the performance of diagnostic tests.

In conclusion, returning travelers are sentinels of the rapidly changing epidemiology of CHIKV; thus, they require a prompt diagnosis and careful surveillance for their possible role in subsequent autochthonous disease transmission. Implementation of user-friendly, rapid, and easily deliverable POC tests for a prompt and accurate laboratory diagnosis is therefore needed to improve patient management and disease control measures.

## References

[1] Kosasih H, Widjaja S, Surya E, Hadiwijaya SH, Butarbutar DP, Jaya UA, Evaluation of two IgM rapid immunochromatographic tests during circulation of Asian lineage chikungunya virus. Southeast Asian J Trop Med Public Health. 2012;43:55–61. 23082554

[2] Rianthavorn P, Wuttirattanakowit N, Prianantathavorn K, Limpaphayom N, Theamboonlers A, Poovorawan Y. Evaluation of a rapid assay for detection of IgM antibodies to chikungunya. Southeast Asian J Trop Med Public Health. 2010;41:92–6. 20578487

[3] Yap G, Pok KY, Lai YL, Hapuarachchi HC, Chow A, Leo YS, Evaluation of chikungunya diagnostic assays: differences in sensitivity of serology assays in two independent outbreaks. PLoS Negl Trop Dis. 2010;4:e753. 10.1371/journal.pntd.0000753 20651930 PMC2907414

[4] Blacksell SD, Tanganuchitcharnchai A, Jarman RG, Gibbons RV, Paris DH, Bailey MS, Poor diagnostic accuracy of commercial antibody-based assays for the diagnosis of acute chikungunya infection. Clin Vaccine Immunol. 2011;18:1773–5. 10.1128/CVI.05288-11 21865416 PMC3187043

[5] Prat CM, Flusin O, Panella A, Tenebray B, Lanciotti R, Leparc-Goffart I. Evaluation of commercially available serologic diagnostic tests for chikungunya virus. Emerg Infect Dis. 2014;20:2129–32. 10.3201/eid2012.141269 25418184 PMC4257799

[6] Okabayashi T, Sasaki T, Masrinoul P, Chantawat N, Yoksan S, Nitatpattana N, Detection of chikungunya virus antigen by a novel rapid immunochromatographic test. J Clin Microbiol. 2015;53:382–8. 10.1128/JCM.02033-14 25411170 PMC4298496

